# Oral Low-Dose Naltrexone in the Treatment of Frontal Fibrosing Alopecia and Lichen Planopilaris: An Uncontrolled Open-Label Prospective Study

**DOI:** 10.7759/cureus.34169

**Published:** 2023-01-24

**Authors:** Remi K Hamel, Ling Chen, Cailin O’Connell, Caroline Mann

**Affiliations:** 1 Dermatology, Washington University School of Medicine, Saint Louis, USA; 2 Biostatistics, Washington University School of Medicine, Saint Louis, USA; 3 Dermatology, Texas A&M College of Medicine, Houston, USA

**Keywords:** lichen planopilaris, frontal fibrosing alopecia, naltrexone, prospective study, open-label, treatment, alopecia

## Abstract

Background

Frontal fibrosing alopecia (FFA) and lichen planopilaris (LPP) is scarring alopecias with limited evidence supporting their treatment options. We investigated the use of low-dose naltrexone (3 mg oral daily) as adjunctive therapy in the treatment of FFA and LPP.

Methods

A single-center, uncontrolled open-label prospective study was performed, with 26 patients who took low-dose naltrexone for one year included in the per-protocol analysis. Both patient-reported (pruritus and burning/pain) and physician-assessed (erythema, scale, and scalp involvement) outcomes were analyzed.

Results

There were decreases in erythema and scale for the overall longitudinal outcomes using linear mixed effects model analysis. However, only erythema had a significant decrease at 12 months compared with baseline. Mean erythema decreased by 0.93 at 12 months compared with baseline on a 0-3-point scale (p<0.0001, 95% mean CI [-1.32, -0.53]). There was no statistically significant difference comparing 12 months to baseline for the other outcomes including pruritus, burning/pain, and scalp involvement. Limitations include the possibility of spontaneous stabilization, concurrent medications, a small sample size with limited racial diversity, and mild subjective symptoms at baseline.

Conclusion

Our study supports further investigation of oral low-dose naltrexone as adjunctive therapy in the treatment of FFA and LPP if there is prominent erythema, and possibly scale.

## Introduction

Lichen planopilaris (LPP) is an inflammatory, cicatricial alopecia, of which frontal fibrosing alopecia (FFA) is considered a clinical variant [1,2]. Features of LPP include perifollicular erythema and scaling leading to the destruction of follicular ostia and patchy scarring alopecia. In FFA, hair loss occurs on the frontotemporal region, often with associated eyebrow loss. Symptoms include pruritus, burning, pain, and tenderness. Treatment options are limited and yield inconsistent results [2]. Topical and intralesional corticosteroids are generally considered first-line [2,3]. Other treatments include oral hydroxychloroquine [4] and doxycycline [2]. Overall, the level of evidence for the above treatments is limited to level IV or V evidence, and further studies into this scarring disease are needed [3,5].

Oral naltrexone is FDA-approved to treat opioid dependence and alcohol use disorder at doses from 50 to 100 milligrams (mg) per day. The multiple mechanisms of low-dose naltrexone (1-5 mg daily) are distinct from high-dose naltrexone [6]. Through intermittent blockade of receptors, low-dose naltrexone causes an increase in endogenous opioids as well as increased expression of μ, δ, and opioid growth factor receptors. Additionally, naltrexone may exert anti-inflammatory effects through the antagonism of toll-like receptor four [7,8]. Low-dose naltrexone has been used off-label with success in the treatment of several dermatologic conditions including LPP, scleroderma, guttate psoriasis, and benign chronic pemphigus [6-9].

A recent review of FFA treatments recommends naltrexone at 3 mg/day in their algorithm, though this is near the bottom of their treatment ladder after the initiation of topical clobetasol/tacrolimus/minoxidil, intralesional triamcinolone, oral finasteride, oral hydroxychloroquine, oral doxycycline, and oral pioglitazone [10]. Another recent review on the treatment of FFA does not mention the use of naltrexone [11]. A case series of four patients with LPP and FFA treated with oral low-dose naltrexone at 3 mg daily reported a reduction of pruritus, clinical evidence of scalp inflammation, and disease progression [2]. Low-dose naltrexone has also been reported as beneficial in relieving symptoms of trichodynia [12]. Based on encouraging data from anecdotal reports and the above case series, we prospectively investigated whether low-dose naltrexone improves patient-reported symptoms, clinical markers of disease activity, and measurement of hair loss progression. This study is also available through ClinicalTrials.gov, NCT04409041.

## Materials and methods

Trial design and oversight

We conducted this single-center uncontrolled open-label prospective study at Washington University in St. Louis (Washington University IRB 201908021 and ClinicalTrials.gov NCT04409041). Enrollment took place between September 1 and December 31, 2019. The study concluded on December 31, 2020. After written informed consent, all participants received oral low-dose naltrexone 3 mg daily for 12 months.

Patients

Adults over 18 years of age were recruited from the pool of patients seen at Washington University Dermatology clinics with clinically or histologically confirmed diagnoses of LPP or FFA. Any baseline severity and duration of disease were allowed. Exclusion criteria were pregnancy, known allergy or hypersensitivity to naltrexone, concurrent opioid use, depression, schizophrenia, or bipolar disorder.

Efficacy and safety assessment

**Figure 1:**
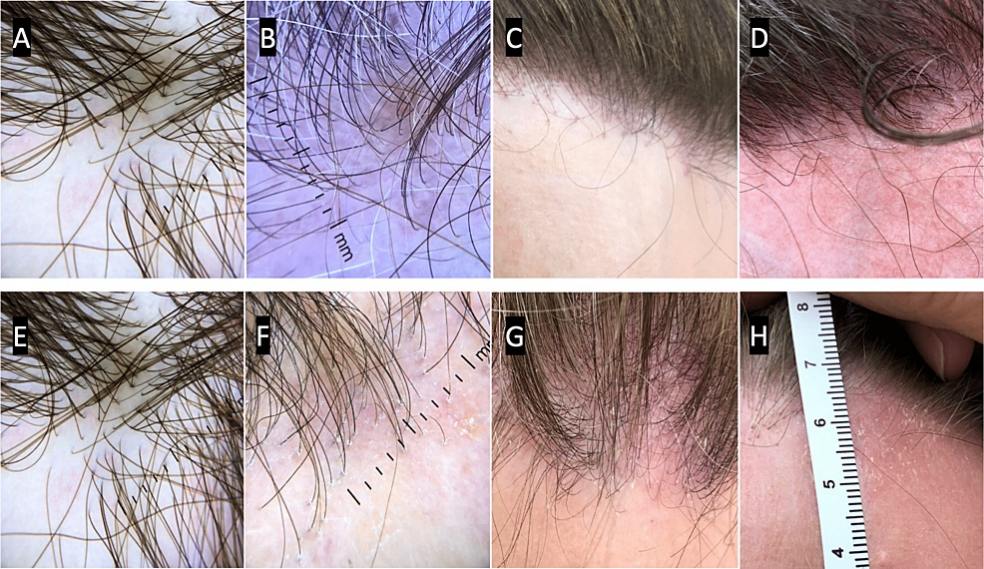
Frontal fibrosing alopecia physician assessment grading scale Panels A-D represent 0-3 erythema, respectively. A) 0 = none, B) 1 = perifollicular pink to red erythema visible only with magnification, C) 2 = perifollicular pink to red erythema visible to naked eye, D) 3 = confluent red erythema visible to the naked eye Panels E-H represent 0-3 scale, respectively. E) 0 = none, F) 1 = perifollicular scale only visible with trichoscope, G) 2 = mild to moderate perifollicular scale visible with the naked eye, H) 3 = areas of confluent thin scale visible with the naked eye

Subjects were seen at baseline and 3, 6, and 12 months in the dermatology clinic of Dr. Caroline Mann. The same investigator (CM) assessed the clinical response to treatment at all visits. At each office visit, data were collected as outlined on the patient assessment form including patient-reported assessments of scalp itching and burning/pain, as well as physical assessments of erythema, scale, and area of scalp involved (Figure 1, Appendix Figure 5). Measurement for scalp involvement was distance (centimeters) from the glabella to the normal frontal hairline (ignoring lone hairs) in FFA and area (centimeters^2^) involved in LPP. Possible adverse effects were recorded at each visit as well as any additional concurrent treatments for FFA or LPP being used. Patients were allowed to continue their other treatments for LPP and FFA during the study period, but no new treatments were added by the dermatologist. The concurrent LPP and FFA medications may have been decreased in dose or discontinued but were not increased in dose.

Statistical methods

Patient characteristics, medications, and side effects were summarized using descriptive statistics. Longitudinal outcomes from time points at zero, three, six, and 12 months were analyzed for each component of the data collection form, including patient assessment of itch and burning/pain (each on a 0-10-point scale), physician assessment for erythema and scale (each on a 0-3 scale) as well as scalp involvement (distance in cm from the glabella to hairline in FFA.) These longitudinal outcomes were analyzed using linear mixed effects model to account for the correlation of measures from the same subject. Time effect was assessed, and linear contrasts were used to compare mean difference between baseline and the other time points. We performed three separate analyses using intention to treat (ITT), modified intention to treat (mITT) and per protocol groups. All statistical tests were two-sided with a significance level of 0.05 and performed with SAS 9.4 (SAS Institute Inc., Cary, NC). 

## Results

Patients

**Figure 2:**
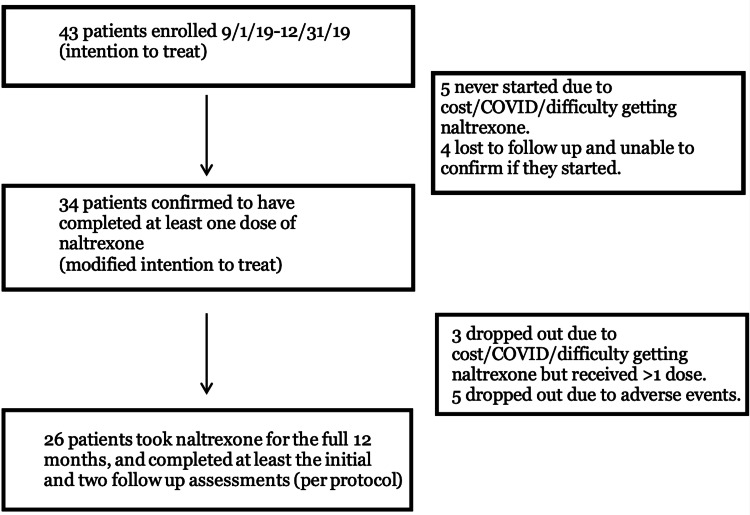
Flow Diagram

Between September 1 and December 31, 2019, a total of 43 patients (ITT population) were enrolled to start 3 mg daily oral low-dose naltrexone. The study concluded on December 31, 2020. 34/43 (79%) of patients were confirmed to have completed at least one dose of naltrexone (mITT population), of which 26/43 (60%) of patients took naltrexone for the full 12 months and completed at least the initial and two follow-up assessments (per protocol population). Patient demographics are shown in Table 1. A flow diagram is shown in Figure 2.

**Table 1:** Patient demographics of the 43 total enrolled patients. FFA: frontal fibrosing alopecia, LPP: lichen planopilaris

Demographics	Result
Age (yr) – mean ± SD	65.0±10.7
Female sex – no. (%)	41 (95.3)
Race – no. (%)	
white	41 (95.3)
black	2 (4.7)
Ethnicity – no. (%)	
non-Hispanic	43 (100)
Diagnosis – no. (%)	
FFA	35 (81.4)
FFA and LPP	2 (4.7)
LPP	6 (14.0)
Biopsy performed – no. (%)	
No	33 (76.7)
Yes	10 (23)

Efficacy

To account for dropouts and missed appointments in the setting of COVID-19, we performed and compared three separate analyses. An ITT analysis was performed on all 43 enrolled subjects. A mITT analysis was performed on 34 patients, excluding the nine patients that never started naltrexone or were lost to follow up after initial assessment and for which it is unknown if they ever received medication. A per-protocol analysis was performed on 26 patients that were confirmed to have taken naltrexone for the full 12 months and had a baseline and at least two follow-up assessments. 

**Figure 3:**
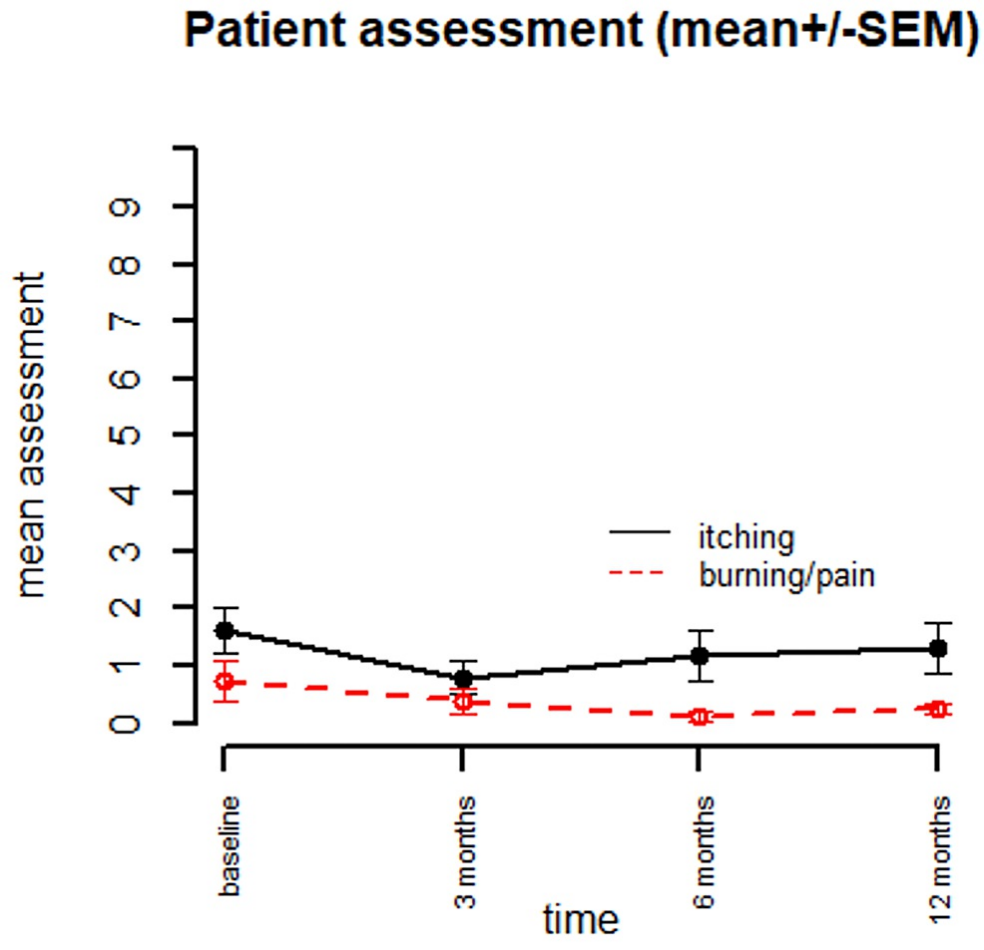
Patient assessment for itching and burning/pain (0-10 scale) SEM: standard error of the mean

**Figure 4:**
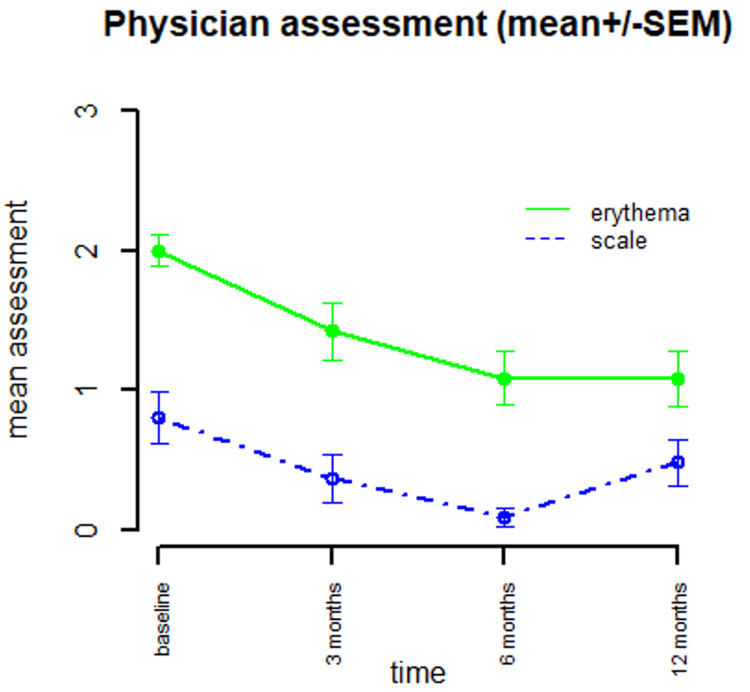
Physician assessment for erythema and scale (0-3 scale) SEM: standard error of the mean

All three analyses were consistent in their results. In a linear mixed model analysis for longitudinal outcomes, there were significant time effects overall for investigator assessments of erythema (ITT p<0.0001, mITT p<0.0001, per protocol p<0.0001) and scale (ITT p=0.0005, mITT p=0.0009, per protocol p=0.01). There was no significant time effect overall for patient reported outcomes of itching (ITT p=0.11, mITT p=0.21, per protocol p=0.28), area involved for FFA only (ITT p=0.28, mITT p=0.31, per protocol p=0.35), or burning/pain (ITT p=0.06, mITT p=0.14, per protocol p=0.11) (Table 2). Of the three analyses which had congruent results, the effect was smallest in the per-protocol analysis. These effects are summarized in Table 3, Figures 3, 4.

**Table 2:** Longitudinal outcome variables over time from per-protocol data.

Mean, median of the longitudinal outcome variables over time (0=baseline, 3=3 month, 6=6 month, 12=12 month). Per protocol data.								
Time (months)	N Obs	Variable	N	Mean	Std Dev	Median	Minimum	Maximum
0	26	ITCHING	26	1.6	2.1	0	0	7
		BURNING_PAIN	26	0.7	1.7	0	0	8
		ERYTHEMA	26	2.0	0.6	2	0	3
		SCALE	26	0.8	0.9	0.5	0	3
3	26	ITCHING	24	0.8	1.4	0	0	6
		BURNING_PAIN	24	0.4	1.2	0	0	5
		ERYTHEMA	19	1.4	0.9	2	0	3
		SCALE	19	0.4	0.8	0	0	2
6	26	ITCHING	24	1.2	2.2	0	0	7
		BURNING_PAIN	24	0.1	0.4	0	0	2
		ERYTHEMA	23	1.1	0.9	1	0	2
		SCALE	23	0.1	0.3	0	0	1
12	26	ITCHING	25	1.3	2.2	0	0	8
		BURNING_PAIN	25	0.2	0.5	0	0	2
		ERYTHEMA	25	1.1	1.0	2	0	2
		SCALE	25	0.5	0.8	0	0	2

**Table 3:** Estimated mean difference from baseline and 95% CI from per-protocol data CI: confidence interval

Outcome	Difference	Estimated mean difference	P-value	95% CI mean difference
Itching	Month 3-baseline	-0.74	0.06	-1.49, 0.02
	Month 6-baseline	-0.48	0.21	-1.23, 0.28
	Month 12-baseline	-0.33	0.38	-1.08, 0.41
Burning/pain	Month 3-baseline	-0.35	0.19	-0.87, 0.18
	Month 6-baseline	-0.61	0.02	-1.14, -0.09
	Month 12-baseline	-0.50	0.06	-1.02, 0.02
Erythema	Month 3-baseline	-0.51	0.02	-0.94, -0.08
	Month 6-baseline	-0.86	< .0001	-1.27, -0.46
	Month 12-baseline	-0.93	< .0001	-1.32, -0.53
Scale	Month 3-baseline	-0.44	0.04	-0.87, -0.02
	Month 6-baseline	-0.71	0.0009	-1.11, -0.30
	Month 12-baseline	-0.33	0.10	-0.72, 0.07

To assess if there was difference in baseline assessments between subjects that did or did not drop out, two sample t-tests were performed. There was no significant difference in baseline characteristics based on dropout status (itching p=0.29, burning/pain p=0.87, erythema p=0.76, scale=0.10, and area involved p=0.31).

We performed a post-hoc power analysis using the per-protocol data because the effect was the weakest in the per protocol group and the per protocol group was also the group of patients that took the low-dose naltrexone and had clinical follow up. Assuming the mean profile and intra-class correlation among within-subject repeated measures as observed, 26 subjects had reached 40.1% and 45.5% power to detect a significant time effect for itching and burning/pain, respectively. To achieve 80% power, 58 and 51 subjects would be needed. The power analysis was based on multivariate linear mixed model analysis with a two-sided F test at a significance level of 0.05. 

Safety

Adverse events were reported in 14/34 (41%) of patients who took at least one dose of naltrexone (Table 4). Vivid dreams were the most reported side effect, reported in 21% of patients, followed by headache, reported by 9% of patients. Five patients dropped out due to adverse events. Adverse events leading to discontinuation included hand stiffness (which on follow up the patient stated was unrelated to naltrexone), headaches, dry mouth, thirst, tongue swelling, and palm itching. One patient dropped out in her first month due to a new diagnosis of breast cancer.

**Table 4:** Reported adverse effects during the low-dose naltrexone study period. Some patients noted at least one side effect so total is greater than 100%.

Adverse events	Number of patients reported	% of total patients (n=34) who took at least one dose of naltrexone
vivid dreams	7	21%
headache	3	9%
breast cancer	2	6%
dry mouth, thirst	1	3%
hyperhidrosis	1	3%
insomnia	1	3%
joint pain	1	3%
metallic taste	1	3%
palm itching	1	3%
sleep disturbance	1	3%
stomach upset	1	3%
tongue swelling	1	3%
vertigo	1	3%
none	20	59%

## Discussion

There are two prior studies using naltrexone for LPP/FFA. Strazzulla et al. reported a case series of four patients with LPP or FFA treated with 3 mg daily of low-dose naltrexone who had a reduction in pruritus and clinical evidence of inflammation. Patients were followed for one, two, two, and seven months, respectively. Three of the four patients were started on pioglitazone at the same time as naltrexone. Patients were also treated with other systemic and topical medications including hydroxychloroquine, doxycycline, finasteride, intralesional triamcinolone, topical clobetasol, tacrolimus, and minoxidil. There were no adverse effects attributed to naltrexone [2]. In a six-month randomized controlled trial of oral low-dose naltrexone (3 mg) versus placebo in patients with lichen planopilaris, the low-dose naltrexone group failed to improve the LPP activity index more than the placebo group. Both groups received topical clobetasol lotion. The only component that differed significantly between treatment groups was scalp erythema (but not perifollicular erythema) [13]. Our finding of reduced erythema is consistent with these two studies. We found a reduction in scale over time, but no significant mean difference between baseline and 12 months. While the effect on the scale was not directly reported in the Strazzulla et al. study, Lajevardi et al. did not find any improvement in scale. Finally, Strazzulla et al. reported improvement in scalp symptoms, but this was not replicated in Lajevardi’s or our studies.

The significance of decreased erythema and scale on overall disease progression is unclear. In a prospective cohort study of 62 patients with FFA, the authors found an association between inflammatory signs and the progression of alopecia, but there was also a group of patients without inflammation that were noted to have disease progression [14]. Other studies have found a more direct correlation. Perifollicular erythema has been correlated with FFA disease activity as measured by the progression of scalp recession [15]. The thickness of the peripilar casts on trichoscopy correlates with the level of inflammatory infiltrate on histopathology [16]. In our study there was no time effect on the FFA area involved as measured from the glabella to the normal hairline, ignoring lone hairs. The frontal hairline was stable in FFA patients during the study period. It is unknown if naltrexone would have any effect on this measurement over a period longer than 12 months.

An LPP assessment index was proposed by Chiang et al. as a weighted composite of patient assessments of pruritus/pain/burning, clinician assessments of erythema/perifollicular erythema/scale, disease spreading, and the anagen pull test [4]. An FFA severity scoring system was proposed by Saceda-Corralo et al. This uses hairline recession, eyebrow loss, perifollicular erythema, and hyperkeratosis, as well as patient pruritus and pain. This was shown to have good intraobserver and excellent interobserver reliability [17]. In our study, we used a modified grading scale using several of these elements and included trichoscopic evaluation.

The cost of naltrexone ($60 per month) was prohibitive for several patients in our study. We could potentially have improved adherence using an alternative dosing method. An alternative method of dosing is to crush ten 50mg tablets of naltrexone (500mg total) into 500 mL of water or juice, thus creating a 1 mg/mL solution. Low-dose naltrexone maintains stability and efficacy through 90 days when crushed into water. At the time of reporting, this solution cost about $15 for a three-month supply [18]. Strazzulla et al. report a typical monthly cost of $35 [2].

In our study, there was no control group to compare side effects. Patients were counseled about vivid dreams, sleep disturbance, and headaches, so this may have introduced bias. In a placebo-controlled randomized trial of low-dose naltrexone in LPP, reported side effects included sleep problems, anxiety, and headache, however the difference between naltrexone and control groups was not statistically significant [13]. Morning dosing of naltrexone has been suggested as a method of mitigating vivid dreams and sleep disturbances [12]. In the absence of signs or symptoms, no specific lab monitoring is required for low-dose naltrexone [7]. A 2019 systematic review and meta-analysis concluded that naltrexone use is not associated with a greater risk of severe adverse events compared with placebo. This was consistent across doses and indications [19].

The annual incidence of female breast cancer is approximately 0.4% in women >50 years old [20]. In our study, 2/34 (5.9%) of patients confirmed to have taken naltrexone developed breast cancer during the study period. Using Epic SlicerDicer, of the 227 patients at Washington University in St. Louis with the diagnosis of FFA from 6/11/20-6/10/21, 12/227 (5.3%) of these FFA patients also had a diagnosis of breast cancer. It is unknown whether patients with FFA are at increased risk for breast cancer and this is an area for further study. Naltrexone has been studied in cancer treatment [21], including in a phase II trial on hormone-refractory metastatic breast cancer (NCT00379197).

Strengths of our study include one-year follow-up and prospective design. Strazzulla et al. reported a mean time for the disease to be stabilized of 10.4 months [22]. Multiple medications are often used concurrently in an attempt to treat LPP/FFA, which can make the interpretation of individual effects challenging. In our study, other medications were used concurrently, but none were started or increased in dose during the trial period.

Limitations of our study include an open-label, uncontrolled, non-randomized, unblinded design. Most of our patients had FFA, and while most authors favor FFA to be a subtype of LPP with similar pathogenesis, the exact relationship is unclear and extrapolation to LPP is limited given the small number of LPP patients in our study. 23% of patients had a biopsy supporting the diagnosis. However, FFA has a distinctive clinical appearance, and most patients with typical clinical findings do not require a biopsy for diagnosis [23]. Our patients with FFA met the diagnostic criteria as proposed by Vañó-Galván et al. and Tolkachjov et al. [24,25]. Heterogeneity in durations of disease and concurrent treatments was not accounted for, and the effect of disease duration and effects of naltrexone together with other treatments is unknown. LPP and FFA are chronic conditions with fluctuating courses [10]. Some effects may have been from this waxing and waning nature. Our sample size was smaller than anticipated due to COVID-19, and thus may underestimate the true effect of naltrexone. Our patients had low baseline itching and burning/pain, so extrapolation to patients with more severe symptoms is limited. Finally, most of our patients were white and female, so generalization to other populations is limited.

## Conclusions

There is limited data supporting the use of low-dose naltrexone in FFA and LPP. We found that the addition of low-dose naltrexone at 3 mg daily modestly improved investigator-assessed outcomes of erythema and, to a lesser extent, scale. There was no significant effect on patient-reported outcomes of pruritus or burning/pain. Low-dose naltrexone can be considered as adjunctive therapy if there is prominent erythema, and this study supports further evaluation of low-dose naltrexone as an adjunctive therapy in cases of LPP/FFA with prominent erythema and scale.

## Figures and Tables

**Figure 5:**
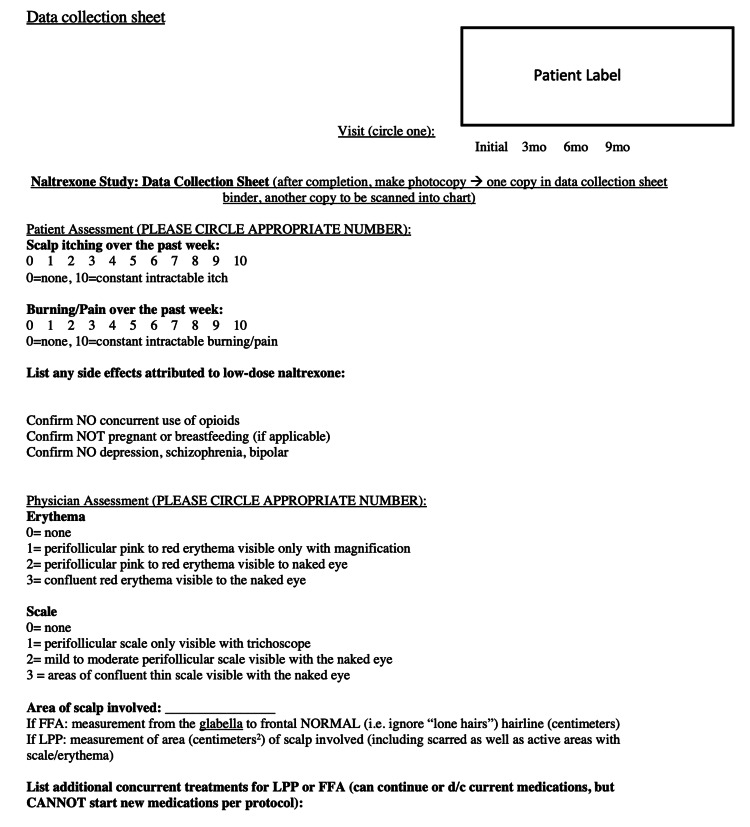
Naltrexone data collection sheet FFA: frontal fibrosing alopecia, LPP: lichen planopilaris

## References

[1] Bolduc C, Sperling LC, Shapiro J (2016). Primary cicatricial alopecia: Lymphocytic primary cicatricial alopecias, including chronic cutaneous lupus erythematosus, lichen planopilaris, frontal fibrosing alopecia, and Graham-Little syndrome. J Am Acad Dermatol.

[2] Strazzulla LC, Avila L, Lo Sicco K, Shapiro J (2017). Novel treatment using low-dose naltrexone for lichen planopilaris. J Drugs Dermatol.

[3] Sperling LC, Nguyen JV (2010). Commentary: treatment of lichen planopilaris: some progress, but a long way to go. J Am Acad Dermatol.

[4] Chiang C, Sah D, Cho BK, Ochoa BE, Price VH (2010). Hydroxychloroquine and lichen planopilaris: efficacy and introduction of Lichen Planopilaris Activity Index scoring system. J Am Acad Dermatol.

[5] Gamret AC, Potluri VS, Krishnamurthy K, Fertig RM (2019). Frontal fibrosing alopecia: efficacy of treatment modalities. Int J Womens Health.

[6] Jaros J, Lio P (2019). Low dose naltrexone in dermatology. J Drugs Dermatol.

[7] Lee B, Elston DM (2019). The uses of naltrexone in dermatologic conditions. J Am Acad Dermatol.

[8] Ekelem C, Juhasz M, Khera P, Mesinkovska NA (2019). Utility of naltrexone treatment for chronic inflammatory dermatologic conditions: a systematic review. JAMA Dermatol.

[9] Sikora M, Rakowska A, Olszewska M, Rudnicka L (2019). The use of naltrexone in dermatology; current evidence and future directions. Curr Drug Targets.

[10] Ho A, Shapiro J (2019). Medical therapy for frontal fibrosing alopecia: a review and clinical approach. J Am Acad Dermatol.

[11] Dina Y, Aguh C (2021). Algorithmic approach to the treatment of frontal fibrosing alopecia: a systematic review. J Am Acad Dermatol.

[12] Tortelly VD, De Mattos T, Fernandes LSA, Nunes BEM, Melo DF (2019). Low-dose naltrexone: a novel adjunctive treatment in symptomatic alopecias?. Dermatol Online J.

[13] Lajevardi V, Salarvand F, Ghiasi M, Nasimi M, Taraz M (2022). The efficacy and safety of oral low dose naltrexone versus placebo in the patients with lichen planopilaris: a randomized controlled clinical trial. J Dermatolog Treat.

[14] Saceda-Corralo D, Pindado-Ortega C, Moreno-Arrones OM, Ortega-Quijano D, Fernández-Nieto D, Jiménez-Cauhe J, Vañó-Galván S (2020). Association of inflammation with progression of hair loss in women with frontal fibrosing alopecia. JAMA Dermatol.

[15] Toledo-Pastrana T, Hernández MJ, Camacho Martínez FM (2013). Perifollicular erythema as a trichoscopy sign of progression in frontal fibrosing alopecia. Int J Trichology.

[16] Martínez-Velasco MA, Vázquez-Herrera NE, Misciali C, Vincenzi C, Maddy AJ, Asz-Sigall D, Tosti A (2018). Frontal fibrosing alopecia severity index: a trichoscopic visual scale that correlates thickness of peripilar casts with severity of inflammatory changes at pathology. Skin Appendage Disord.

[17] Saceda-Corralo D, Moreno-Arrones ÓM, Fonda-Pascual P (2018). Development and validation of the frontal fibrosing alopecia severity score. J Am Acad Dermatol.

[18] Bronfenbrener R (2021). Inexpensive compounding of low-dose naltrexone (LDN) with orange juice. J Am Acad Dermatol.

[19] Bolton M, Hodkinson A, Boda S (2019). Serious adverse events reported in placebo randomised controlled trials of oral naltrexone: a systematic review and meta-analysis. BMC Med.

[20] Ravdin PM, Cronin KA, Howlader N (2007). The decrease in breast-cancer incidence in 2003 in the United States. N Engl J Med.

[21] Couto RD, Fernandes BJ (2021). Low doses naltrexone: the potential benefit effects for its use in patients with cancer. Curr Drug Res Rev.

[22] Strazzulla LC, Avila L, Li X, Lo Sicco K, Shapiro J (2018). Prognosis, treatment, and disease outcomes in frontal fibrosing alopecia: a retrospective review of 92 cases. J Am Acad Dermatol.

[23] Iorizzo M, Tosti A (2019). Frontal fibrosing alopecia: an update on pathogenesis, diagnosis, and treatment. Am J Clin Dermatol.

[24] Vañó-Galván S, Saceda-Corralo D, Moreno-Arrones ÓM, Camacho-Martinez FM (2018). Updated diagnostic criteria for frontal fibrosing alopecia. J Am Acad Dermatol.

[25] Tolkachjov SN, Chaudhry HM, Imhof RL, Camilleri MJ, Torgerson RR (2018). Reply to: "updated diagnostic criteria for frontal fibrosing alopecia". J Am Acad Dermatol.

